# Whole Blueberry and Isolated Polyphenol-Rich Fractions Modulate Specific Gut Microbes in an In Vitro Colon Model and in a Pilot Study in Human Consumers

**DOI:** 10.3390/nu12092800

**Published:** 2020-09-12

**Authors:** Alexandra Ntemiri, Tarini S. Ghosh, Molly E. Gheller, Tam T. T. Tran, Jamie E. Blum, Paola Pellanda, Klara Vlckova, Marta C. Neto, Amy Howell, Anna Thalacker-Mercer, Paul W. O’Toole

**Affiliations:** 1 School of Microbiology, University College Cork, T12 K8AF Cork, Ireland; alexandra.ntemiri@ucc.ie (A.N.); tarini.ghosh@ucc.ie (T.S.G.); tam.tran@ucc.ie (T.T.T.T.); paola.pellanda@ucc.ie (P.P.); klara.vlckova@ucc.ie (K.V.); marta.neto@ucc.ie (M.C.N.); 2 APC Microbiome Ireland, University College Cork, T12 K8AF Cork, Ireland; 3 Marucci Center for Blueberry Cranberry Research, Rutgers University, Chatsworth, NJ 08019, USA; ahowell@njaes.rutgers.edu; 4 Division of Nutritional Sciences, Cornell University, Ithaca, NY 14853, USA; meg369@cornell.edu (M.E.G.); jeb462@cornell.edu (J.E.B.); aet74@cornell.edu (A.T.-M.); 5 Department of Cell, Developmental and Integrative Biology, University of Alabama at Birmingham, Birmingham, Alabama, AL 35294, USA

**Keywords:** polyphenols, blueberries, gut microbiota, in vitro, human study, oxidative stress

## Abstract

Blueberry (BB) consumption is linked to improved health. The bioconversion of the polyphenolic content of BB by fermentative bacteria in the large intestine may be a necessary step for the health benefits attributed to BB consumption. The identification of specific gut microbiota taxa that respond to BB consumption and that mediate the bioconversion of consumed polyphenolic compounds into bioactive forms is required to improve our understanding of how polyphenols impact human health. We tested the ability of polyphenol-rich fractions purified from whole BB—namely, anthocyanins/flavonol glycosides (ANTH/FLAV), proanthocyanidins (PACs), the sugar/acid fraction (S/A), and total polyphenols (TPP)—to modulate the fecal microbiota composition of healthy adults in an *in vitro* colon system. In a parallel pilot study, we tested the effect of consuming 38 g of freeze-dried BB powder per day for 6 weeks on the fecal microbiota of 17 women in two age groups (i.e., young and older). The BB ingredients had a distinct effect on the fecal microbiota composition in the artificial colon model. The ANTH/FLAV and PAC fractions were more effective in promoting microbiome alpha diversity compared to S/A and TPP, and these effects were attributed to differentially responsive taxa. Dietary enrichment with BB resulted in a moderate increase in the diversity of the microbiota of the older subjects but not in younger subjects, and certain health-relevant taxa were significantly associated with BB consumption. Alterations in the abundance of some gut bacteria correlated not only with BB consumption but also with increased antioxidant activity in blood. Collectively, these pilot data support the notion that BB consumption is associated with gut microbiota changes and health benefits.

## 1. Introduction

The gut microbiota is a recognized modulator of human health and is shaped by host genetics, environment, lifestyle, and diet [1,2]. Most studies investigated cohorts representing western populations and lifestyle and compared healthy subjects and individuals with diverse conditions. Despite significant inter-individual variations in the gut microbiota composition, a general description of the healthy adult gut microbiota has emerged [3], but the variation range of phylum proportional abundances in healthy subjects is still large. Factors working throughout the lifespan such as repeated antibiotic use, significant changes in dietary habits, and infections may lead to perturbations and reductions in the composition and phylogenetic diversity of the gut microbiota that are associated with disease [4].

Immuno-senescence, hospitalization, and changes in dietary habits may collectively contribute to the age-related gut microbiota alterations observed in older individuals and that are linked in turn to the increase in the inflammatory state of older adults, a known risk factor for mortality in humans and animal models [5,6]. In conditions characterized by an altered microbiome at any point in life, gut microbiota manipulation can be a target for prevention, improvement, or even therapy [7,8]. This could be achieved with the use of probiotics, fecal microbiota transplants (FMT), live biotherapeutics, or prebiotics [9]. Accumulating evidence suggests that polyphenols are a dietary component with potential prebiotic activity [10,11].

Polyphenols are plant-derived dietary components that can be grouped into non-flavonoids and flavonoids [12]. Non-flavonoids include compounds such as tannins, phenolic acids, and lignans [12]. Flavonoids include isoflavones; neoflavonoids; and others such as chalcones, flavones, and flavonols, which are the building blocks of proanthocyanidins and anthocyanins [13]. Anthocyanins are present in plants as glycosylated anthocyanidins conjugated with sugars including glucose, galactose, arabinose, rhamnose, and xylose [14]. The dietary intake of these compounds has been associated with health benefits based on in vitro and in vivo experimental models and human studies [10,15].

The levels of flavonoid consumption reported for adult populations vary significantly, likely due at least in part to differences in the analytical methods and associated reference standards used to assess the flavonoid content in food products, but also because of widely varying dietary habits [14,16,17]. Adults in the US, Europe, and the UK have a daily consumption of flavonoids that ranges from 177 mg/d up to 428 mg/d, and a consumption of anthocyanidins that ranges from 4.2 mg/d up to 19 mg/d [16,18,19].

Unabsorbed phenolic compounds reach the colon, where they may serve as substrates for fecal microbiota fermentation [20]. Several *in vitro* [21,22], *in vivo* [23,24,25,26,27], and human studies [28,29,30] indicate health benefits and the potential for polyphenols to modulate the gut microbiota. There is also scientific interest in the combined effect of dietary polyphenols and fiber on the gut microbiota [31]. In this context, blueberry (BB) consumption may provide adequate amounts of dietary polyphenols with potential health benefits [32].

To further elucidate the interactions between BB ingredients and the human gut microbiome, we profiled the compositional changes that occurred in batch fermentations inoculated with the fecal microbiota of healthy young adults and supplemented with isolated BB polyphenol-rich fractions. These fractions are individually enriched for different major classes of presumptive bioactive BB ingredients, and have been tested for their activity in multiple previous publications [33,34,35,36,37]. To detect microbiota taxa that are responsive to human BB consumption, 17 healthy female volunteers in two age groups consumed freeze-dried BB for 6 weeks, and their gut microbiota composition was analyzed before and after the dietary intervention. The data indicate that BB ingredients or whole BB fruit can affect the microbiota both in *in vitro* colon model systems and in human consumers, but the effects on microbiota diversity are greater in older consumers.

## 2. Materials and Methods

### 2.1. Faecal Inocula and the In Vitro Colon Model

A pool of 5 fecal microbiota samples was used to inoculate the fermenter vessels comprising an artificial colon model. The five fecal samples were collected from healthy young donors (coded as follows: HYD3 32 years old (yrs), HA4 26 yrs, HA6 29 yrs, HA7 29 yrs, HA8 35 yrs) under a procedure approved by the local clinical research ethics committee. All the subjects gave their informed consent for inclusion before they participated in the study. The study was conducted in accordance with the Declaration of Helsinki, and the protocol was approved by the Ethics Committee/Institutional Review Board at Cornell University (Protocol ID#: 1706007263).

Fecal samples were collected and transferred to an anaerobic cabinet no later than one hour after passing. Each fecal sample was homogenized in a reduced sterile solution of PBS containing 20% glycerol and stored at −80 °C. Before each fermentation run, an aliquot of each of the 5 fecal samples (i.e., microbiota samples) was thoroughly thawed in an anaerobic cabinet and mixed in equal volumes for the inoculum.

Batch fermentations were used to simulate the colonic bacterial fermentation of the selected substrates [38]. One percent (*w*/*v*) fecal inoculum was prepared to inoculate each of three parallel single vessels with a 150 mL working volume in each vessel. A continuous flow of NO_2_ was used to maintain anaerobic conditions during the 24 h pH (6.8) and temperature (37 °C)-controlled fermentation runs, with continuous stirring and atmosphere monitoring. Adaptation to in vitro media results in reduced microbiota diversity, as observed in previous studies [39]. To reduce this loss of microbiota diversity, the basal fermentation medium was supplemented with a mix of prebiotic fibres (referred to as MIX) (xylan 2 g/L; pectin 2 g/L; arabinogalactan 2 g/L; soluble starch 4 g/L) plus amylopectin (1 g/L), beta glucan (0.5 g/L), and glucose (2 g/L) [40,41].

The MIX medium was supplemented separately with each of 4 different BB polyphenol fractions: i. anthocyanins/flavonol glycosides (200 mg/L); ii. proanthocyanidins (200 mg/L); iii. sugar/acid fraction; and iv. total polyphenolics (333 mg/L). These were prepared as described in Section 2.4. A fermentation run without any supplementation was performed as a control. Samples from the fermentation culture were retrieved at 0, 16, and 24 h and centrifuged immediately, and the pellet and supernatants were kept at −20 °C for further analysis.

### 2.2. Bacterial DNA Extraction for In Vitro and Human Study

A 200 mg quantity of fecal pellet was weighed as instructed in the QIamp Fast DNA Stool (Qiagen, Manchester, UK) extraction kit protocol. The samples were homogenized mechanically in sterile tubes containing InhibitEX solution and zirconia glass beads of three sizes—0.1, 0.5, and 1.0 mm (Thistle Scientific Ltd., Glasgow, UK)—using a Mini-Beadbeader (Biospec Products, Inc., Bartlesville, OK, USA). The subsequent steps of gDNA extraction were performed as previously described by our laboratory [39].

### 2.3. Microbiome Profiling of In Vitro and Human Study Samples

The primers S-D-Bact-0341-b-S-17 (5′-CCTACGGGNGGCWGCAG-3′)/S-D-Bact-0785-a-A-21 (5′GACTACHVGGGTATC TAATC C-3′) 5′ [42] were used to amplify the V3/V4 variable region of the 16S rRNA gene for the profiling of the bacterial fecal microbiota using the Phusion High-Fidelity PCR Master Mix (ThermoFisher Scientific, Waltham, MA, USA). After PCR product purification, the Illumina MiSeq system protocol was used for library preparation. Indexing PCR was performed to amply the dual-index barcodes to the amplicon (Nextera XT V.2 Index Kits; Illumina, San Diego, CA, USA). The purification of the barcoded amplicons was performed with the Agencourt AMPure XP-PCR Purification system (Beckman Coutler, Inc., Brea, CA, USA). The Qubit dsDNA HS Assay Kit (Thermo Fischer Scientific, Waltham, MA, USA) was used to quantify the products. Equal concentrations of all the purified amplicons were pooled into a library that was sequenced (2 × 300 bp) on a MiSeq Illumina platform in the Teagasc Food Research Centre sequencing facility (Teagasc Moorepark, Fermoy, Ireland). ENA accession number: PRJEB39031.

### 2.4. Isolation of Enriched Fractions for Bioassay

Four major ingredient fractions of BB were prepared essentially as previously described in several previous publications [32,33,34,35,36,37]. In detail, whole frozen BB, cv. “Coville”, were extracted, and fractions of total polyphenolics (TPP), proanthocyanidins (PACs), anthocyanin/flavonol glycosides (ANTH/FLAV), and sugars/acids (S/A) were isolated using solid-phase chromatography according to Howell et al., 2005 [43]. Briefly, BBs were homogenized with water in a blender and applied to a C18 column (Waters Corp., Milford, MA, USA) preconditioned with MeOH followed by dH_2_O. The S/A fraction was collected as the column was washed with dH_2_O then dH_2_O:MeOH (85:15) (*v*/*v*), followed by elution with acidified aqueous methanol. Solvents were removed from the S/A fraction under reduced pressure. The TPP fraction containing anthocyanins, flavonol glycosides, and PACs (confirmed using reverse-phase HPLC with diode array detection) was eluted with 1% HOAc in MeOH (*v*/*v*). All the fractions were dried under reduced pressure to remove the solvent. The TPP fraction was then suspended in 50% EtOH, and applied to a Sephadex™ LH-20 (Sigma Chemical Co., St. Louis, MO) column that was pre-equilibrated overnight in EtOH:dH_2_O (50:50) (*v*/*v*). The ANTH/FLAV fraction was eluted with 50% EtOH and dried to remove the solvent. The PAC fraction was eluted from the LH-20 column with 70% aqueous acetone, and monitored for purity using diode array detection at 280 nm. The absence of absorption at 360 nm and 450 nm confirmed that flavonol glycosides and anthocyanins, respectively, were removed. Acetone was evaporated under reduced pressure, and the resulting purified PAC fraction was dried. Analytical tools, including mass spectrometry and NMR spectrometry, have been routinely utilized to confirm the composition of these BB fractions using the method of Schmidt et al. (2004) and others [44,45,46].

### 2.5. Human Study Design

This study was approved by the Cornell University institutional review board and complies with the Helsinki Declaration. This trial is registered at clinicaltrials.gov (NCT04262258). All the participants provided written informed consent prior to participation in the study.

Seventeen healthy young (aged 21–39 yrs, *n* = 11) and old (aged 65–77 yrs, *n* = 6) women participated in the study. Potential participants were screened using an online survey to assess eligibility. After initial eligibility was established, the participants came to the Human Metabolic Research Unit at Cornell University to complete a health history questionnaire and provide information on current and recent medications. Inclusion criteria were females between the ages of 21 and 40 yrs y and 60 and 79 yrs. Participants were excluded if they had a musculoskeletal disease (e.g., rheumatoid arthritis) or other disorder that would impact skeletal muscle function (e.g., diabetes or cancer), were taking immunosuppressive medication, were pregnant or breastfeeding, had a high alcohol intake (>11 drink per week), had an allergy or intolerance to blueberries, and had antibiotic use within the past 6 months.

After the participants were enrolled in the study, they began a 2-week washout period in which they were asked to avoid foods rich in polyphenols and anthocyanins. Following the 2-week washout period, the participants began the blueberry enriched diet (BED); the participants were instructed to consume 38 g (two packages of 19 g) of freeze-dried BBs (*Vaccinium virgatum* (ashei)/*Vaccinium corymbosum*) with water daily for 6 weeks. Compliance was monitored through a supplement compliance log and empty BB packets returned by the participants to the study personnel.

Fasting stool and blood samples were obtained at four time points (washout, week 2, week 4, and week 6) throughout the BED study. The participants were given pre-labelled stool sample collection kits, and samples were collected outside of the lab. The participants transported samples to the lab with an insulated bag that contained an ice pack. The samples were immediately placed in −80 °C freezer until they were processed. To obtain plasma, venous blood samples (~10 mL) were collected in ethylenediaminetetraacetic acid (EDTA) tubes (Becton Dickinson Vacutainer system; Becton Dickinson, Franklin Lakes, NJ, USA) and then immediately centrifuged (4 °C at 1200× *g* for 10 min) to obtain plasma. Plasma was transferred to a new tube (volume ~500 uL) and stored in a −80 °C freezer until all the participants had completed the study and the samples were ready for analysis.

The participants BED_001, 002, 003, 006, 007, 008, 009, 011, 013, 015, and 016 belonged to the young age group and the participants BED_004, 005, 010, 014, 018, and 020 belonged to the older age group (Table 1).

### 2.6. Plasma FRAP Assay

The ferric-reducing antioxidant power (FRAP) was determined in plasma from the BED donors following established methods [47]. Briefly, a solution of sodium acetate (EMDMillipore, Burlington, MA, USA), 2,4,6 tripyridyl-S-triazine (TPTZ, ACROS Organics, Geel, Belgium), and ferric chloride (Fisher Scientific, Waltham, MA, USA) was incubated with plasma samples or ferrous sulfate (assay standard, Fisher Scientific, Waltham, MA, USA) for 4 min at 37 °C. The absorbance was measured at 593 nm and standardized to the absorbance of the ferrous sulfate standard to derive the FRAP value (μmol/L). Four technical replicates were measured. The absorbance of the blank was subtracted from each measurement, and the FRAP value of each sample was determined using the following formula: (sample absorbance)/(assay standard absorbance) * (assay standard concentration (1000 μmol/L)).

### 2.7. Plasma Glucose and CRP Measurements

Routine panels were conducted on stored fasting plasma samples (glucose and C-reactive protein) at Cornell University’s Human Nutritional Chemistry Service Laboratory. Glucose was analyzed on a Dimension Xpand chemistry analyzer (Siemens Healthineers, Malvern, PA, USA), and CRP was measured on an Immulite 2000 immunoassay system (Siemens Healthineers, Malvern, PA, USA).

### 2.8. Microbiota Composition and Statistical Analysis

The pipeline for the microbiota composition analysis was described before [48] and comprised the following steps. Paired-end reads were joined with FLASH [49] and quality filtering was performed in Qiime (v.1.9.1) using the split_libraries_fastq.py script [50]. The forward and reverse primers were removed using cutadapt [51] and the script truncate_reverse_primer.py, respectively. For additional quality filtering and de novo operational taxonomic unit (OTU) clustering, USEARCH was used. Filtering by length and size was performed before single unique sequences were excluded. Clustering in OTUs was performed using 97% identity for the sequences after the various filtering steps. Chimeras were removed based on the use of UCHIME with the GOLD reference database. The OTUs were used to map sequences initially filtered for quality (97% identity). The mothur suite of tools (v1.36.1) and the RDP (trainset 14) were used for the OTU classification (classify.seqs) with a 80% confidence threshold [52]. The OTUs were classified down to the species level with SPINGO [53].

The PyNast tool [54] in Qiime (along with the diversity function of the vegan package version 2.4.3 of the R programming interface v 3.5.4) was used to align the sequences and calculate the alpha (α) diversity indices—i.e., Shannon, Simpson, Chao1, Phylogenetic Diversity (PD), Observed Species, and beta (β) diversity indices (i.e., Weighted Unifrac and Unweighted Unifrac). Weighted Unifrac and Spearman distances were used for principal coordinates analysis (PCoA) (ade4 package) using the R programming interface (v 3.5.4). PERMANOVA analysis was performed using the adonis function implemented in the vegan package (version 2.4.3) of the R programming interface. The reads assigned to taxa at various levels (OTU, species, and genus) were cumulated and divided by the total number of reads per sample.

For the artificial colon reactor analysis, the differentially abundant taxa in the different supplementations were identified by Kruskal–Wallis H-test followed by Dunns’ test, and the *p* values were adjusted for multiple testing by Benjamini Hochberg (BH) (*p*_adj_). Significant results were indicated with * (*p*_adj_ < 0.05), and marginal differences with # (*p*_adj_ < 0.1). The dunn.test package of the R programming interface was used for this purpose (run with the method = “bh” argument to specify adjustment procedure to Benjamini Hochberg-BH). For this analysis, supplementation-specific abundances of the various taxa at 16 h and 24 h were combined. For each of these taxa, we also checked whether their abundances exhibited significant variations in their abundances at 16 h and 24 h using Wilcoxon Signed Rank Tests. The same approach (as described above) was also adopted for testing differences in the α diversity measures. The microbiota profiles resulting from the supplementation regimes were grouped into two groups (G1 and G2), as described in the results. The “Within G1” and “Within G2” distances were obtained as follows. For each microbiota resulting from a given supplementation, the median of the Weighted Unifrac distances of the microbiota with all the other microbiotas belonging to the same supplementation (that is, all the other microbiotas belonging to either G1 or G2 at 16 h and 24 h) was obtained. These median distances represented the microbiota variations within that given supplementation group. For the “Across G1 and G2” variations, for each sample belonging to a given supplementation, the median of the Weighted Unifrac distances of the microbiota with all the other microbiotas belonging to the other supplementations (that is, all the other microbiotas belonging to either G1 or G2 at 16 h and 24 h) was obtained. The wilcox.test function of the R programming interface v 3.5.4 was then used for comparing the within and across-group median distances.

For the human study, the OTU co-abundance groups (CAGs) demonstrating similar mean abundance pattern trends across time points were identified. The identification of taxa CAGs and their distinct taxonomic/temporal abundance profiles was conducted as follows. The mean abundance of each OTU was obtained for W0, W2, W4, and W6, providing a mean temporal trend of each OTU across time points. Subsequently, the Kendall correlations (taus) across all pairs of OTUs were obtained and then converted to Kendall distances. The Kendall distance between any two pairs of OTUs was calculated as 1—(Kendall tau)/2. The OTUs were clustered into CAGs based on their mutual abundance pattern (Ward-D2 method). The heatmap.2 function of R v 3.5.4 was used to visualize the clustering of the CAGs with the colors assigned using the RColorBrewer function. The Kruskal–Wallis H test followed by Dunns’ test was used for a CAG abundance comparison across time points (using the dunn.test function, as described earlier for the differentially abundant taxa analysis in colonic reactor models). The OTUs were classified using SPINGO (0.65 threshold). For each CAG, OTUs with defined species classifications were obtained, and the frequency of each species in a given CAG was computed. The representation of the different species in the various CAGs were depicted using word clouds using the “wordcloud” module of the R programming interface v 3.5.4. A PERMANOVA analysis (Spearman distances at OTUs and CAG abundances level) was performed for the association between the taxa abundances (OTU and CAG level) and CRP, glucose, and FRAP.

For the FRAP values, the associations were further validated using Random Forest models (using the combination of the rfcv and randomForest functions of the randomForest module of R v 3.5.4). A total of 100 iterations of the Random Forest models were performed, each time taking 50% of the samples for training and testing on the other 50%. For each sample, the mean predicted FRAP values were then correlated with the actual FRAP values to ascertain the strength of the association. A key advantage of the Random Forest models is that they can not only be used to predict a given trait (either quantitative or categorical; in this case, the quantitative FRAP values) from a dataset of multiple predictor features (in this case, the OTUs), but they can provide the relative importance of each of the features to predict the given trait. This enables the identification of the most optimal set of associated features for predicting a given trait. Using these scores, the core list of FRAP-positive and FRAP-negative marker OTUs was identified, as described in detail in the Results section. The enrichment of the different CAGs in the two FRAP-associated marker OTUs was observed using Fishers’ exact test. Spearman correlations (and the associated *p* values) were computed using the corr.test function of the psych package of the R programming interface (run with the adjust = “fdr” argument for p value correction-false discovery rate FDR). The volcano plots showing the positive and negative associations of the various taxa with the clinical metadata were using the ggplot and ggrepel modules of R v 3.5.4.

## 3. Results

### 3.1. A Prebiotic MIX, PACs, or ANTH/FLAV Have a Similar Effect on Microbiota Structure in the In Vitro Colon Model

**Figure 1:**
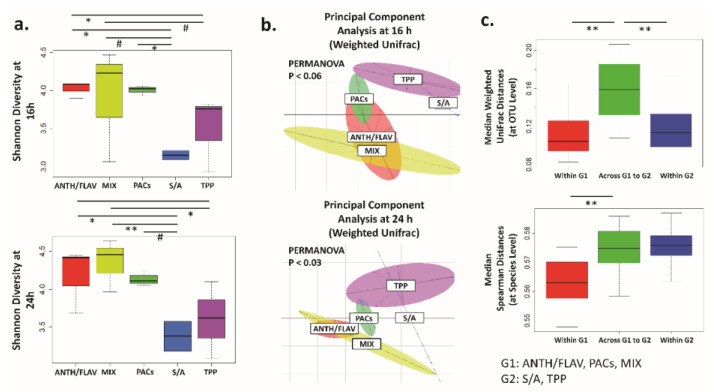
Differential fecal microbiota structure patterns due to the blueberry (BB) polyphenol-rich fraction supplementation in the *in vitro* colon model. (**a**). Boxplots showing the Shannon α diversity index in different supplementation regimes at 16 h and 24 h. (**b**). Principal Components Analysis (PCoA) based on the weighted Unifrac distances of the fecal microbiota for 16 h and 24 h *in vitro* fermentations. PERMANOVA *p* values for each time point are indicated. (**c**). Boxplots showing the microbiota variation within in each group “Within G1” and “Within G2” (16 h and 24 h microbiotas combined) and “Across G1 to G2”. Horizontal bar plots highlight the significant differences across the supplementation regimes: * *p*_adj_ < 0.05; ** *p*_adj_ < 0.01. Marginal differences are also noted: # *p*_ad__j_ < 0.10. ANTH/FLAV: anthocyanin/flavonols glycoside supplementation; MIX: prebiotic fibers mix supplementation; PACs: proanthocyanidins supplementation; S/A: sugar/acid fraction supplementation; TPP: total BB polyphenols.

The fecal microbiota α diversity (Shannon and Observed Species) was reduced over the 24 h of fermentation (observed at the 16 h and 24 h time points) compared to the baseline 0 h (Supplementary Materials Figure S1), which is a typical feature of in vitro colon models [43]. Supplementation with ANTH/FLAV, PACs, and prebiotic MIX resulted in microbiota communities displaying similar Shannon and Observed Species diversity index values that were noticeably higher than in the fermentations with either S/A and TPP supplementation (significant for PACs/MIX versus S/A with *p*_adj_ < 0.5) (Figure 1a; Figure S2). Thus, in the in vitro colon model used in this study, isolated BB components such as the polyphenol-rich fractions ANTH/FLAV and PACs were more efficient in promoting microbiota diversity than the other polyphenol-rich fractions used in this study—i.e., TPP or the S/A fraction.

To visualize the global effect of supplementing the fecal fermentation with BB polyphenol-rich fractions, we performed Principal Coordinate Analysis (PCoA) based on weighted and unweighted Unifrac distance measures (Figure 1b; Figure S3). Unweighted Unifrac analysis, where the taxa presence/absence is taken into account, showed that TPP supplementation led to a separation of the microbiota at 16 h that was not sustained through the 24 h (Figure S3). Weighted Unifrac analysis, in which the abundance of dominant taxa is more impactful on β diversity measurement, showed that TPP and S/A supplementation led to a microbiota profile separate from that of the other supplementation regimes at both 16 h (marginal but not significant variation; PERMANOVA *p* < 0.06) and 24 h (significant; PERMANOVA *p* < 0.03) (Figure 1b). Supplementation with the ANTH/FLAV and PACs fractions resulted in a microbiota β diversity close to that promoted by MIX (Figure 1b). Thus, the BB polyphenols tested had distinct effects on the fecal microbiota. The PCoA analysis showed that the supplementations could be grouped into G1, consisting of ANTH/FLAV, MIX, and PACs; and G2, consisting of S/A and TPP. The relatedness of samples within G1 and G2 and across G1 and G2 are shown in Figure 1c.

**Figure 2:**
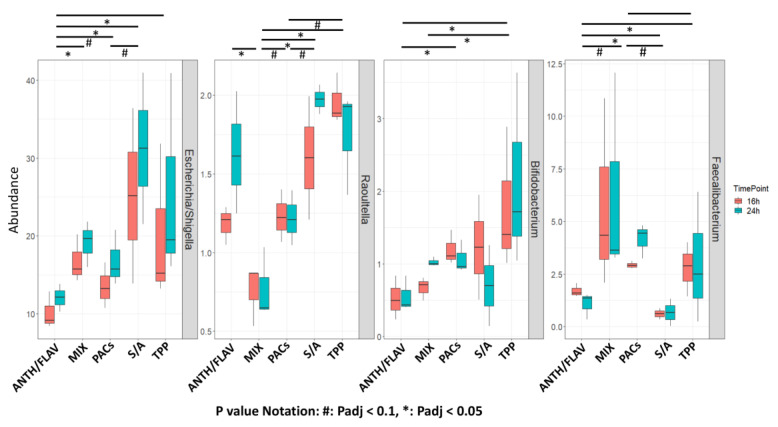
Differentially abundant taxa (genus level) in the fecal microbiota after *in vitro* supplementation with blueberry (BB) polyphenol-rich fractions. The results of relative abundance (>1%) from 16 h and 24 h fermentations are shown in pink and green color, respectively. Significant differences for comparisons combining the 16 h and 24 h data (post-hoc Dunn’s test with Benjamini-Hochberg (BH) *p*_adj_) between the corresponding pairs per supplementation across supplementations are indicated in horizontal bas plots: * *p*_adj_ < 0.05. Marginal differences are also indicated: # *p*_adj_ < 0.10. ANTH/FLAV: anthocyanin/flavonol glycoside supplementation; MIX: prebiotic fibers mix supplementation; PACs: proanthocyanidin supplementation; S/A: sugar/acid fraction supplementation; TPP: total BB polyphenols.

The differences in α diversity observed between ANTH/FLAV, PACS, and MIX and TPP and S/A supplementation could be partially attributed to certain taxa dominating in relative abundance in the microbiota at 16 h and 24 h; non-significant microbiota differences were observed between the two time points per supplementation (Table S1; Figure S4). The supplementation-specific differences in various taxa were investigated by first performing a descriptive analysis and comparison of the supplementation-specific taxa at the family level (Figure S4), followed by a statistical comparison of the taxa abundances (genus and family level) (across supplementation combining the 16 h and 24 h time points) (Figure 2; Figure S5). The decrease in α diversity observed in the fecal microbiota fermented with TPP and S/A supplementation could be explained by the comparatively higher abundance of *Enterobacteriaceae* (49.77% and 47.49% average relative abundance, respectively) observed by compositional analysis of the fecal microbiota after 16 h and 24 h of fermentation (Figure S4). The lowest *Enterobacteriaceae* abundance was observed upon ANTH/FLAV supplementation and MIX (average relative abundance of 31.46% and 29.11%, respectively) (Figure S4). ANTH/FLAV supplementation resulted in a significantly lower *Escherichia*/*Shigella* (*Enterobacteriaceae*) relative abundance compared to the prebiotic MIX (*p*_adj_ < 0.05), S/A fraction (*p*_adj_ < 0.05), and TPP (*p*_adj_ < 0.05) supplementation (Figure 2). *Lachnospiraceae* was a major microbiota family that was reduced in abundance compared to baseline (34.4% average relative abundance) across fermentation regimes, potentially due to the in vitro conditions (Figure S4). Supplementation with ANTH/FLAV, PACs, or prebiotic MIX sustained the highest *Lachnospiraceae* (average relative abundance of 14.52%) in the microbiota (Figure S4). Similarly, the *Bacteroidaceae* relative abundance was higher upon ANTH/FLAV, PACs, and prebiotic MIX supplementation (16.61%, 15.1%, and 12.95% average relative abundance, respectively), and overall increased in abundance from baseline (average relative abundance of 6.89%) across supplementations (Figure S4).

The abundance of the health-relevant genus *Bifidobacterium* spp. (*Bifidobacteriaceae*; 2.54% average abundance at 0 h (Figure 2; Figure S4) was significantly increased upon TPP supplementation (1.95% average relative abundance) compared to MIX (0.85% average relative abundance; *p*_adj_ < 0.05) and ANTH/FLAV supplementation (0.54% average relative abundance; *p*_adj_ < 0.05) (Figure 2). The health-relevant taxon *Faecalibacterium* was present at an average relative abundance of 6.04% (median abundance of 4%) upon MIX supplementation, which was marginally higher compared to PACs (*p*_adj_ < 0.1), and significantly higher compared to the TPP (*p*_adj_ < 0.05) and S/A (*p*_adj_ < 0.05) fraction supplementation (Figure 2).

Apart from the aforementioned supplementation effects on the dominant taxa, the low abundance taxa (<1% average relative abundance) were also differentially abundant in the microbiota depending on the supplementation of the fermentation medium (Figure S5). Supplementation with ANTH/FLAV resulted in an increased relative abundance of *Phascolarctobacterium* compared to MIX (significant; *p*_adj_ < 0.05), S/A (marginal; *p*_adj_ < 0.1) and TPP (significant; *p*_adj_ < 0.05) supplementation, and of *Gemmiger* compared to MIX and PACs (*p*_adj_ < 0.05 for both supplementations). The *Clostridium* cluster XIVb relative abundance was increased with PACs supplementation (*p*_adj_ < 0.05 compared to ANTH/FLAV, S/A, and TPP). The *Sutterella* relative abundance was increased upon ANTH/FLAV supplementation (*p*_adj_ < 0.05 compared to S/A and TPP). The *Oscillibacter* and *Flavonifractor* relative abundance was significantly increased with MIX and PACs supplementation, whereas the *Burkholderiales* relative abundance was highest after the PAC supplementation (significantly higher: *p*_adj_ < 0.05 compared to TPP; marginally higher: *p*_adj_ < 0.1 as compared to ANTH/FLAV) (Figure S5). The unclassified *Erysipelotrichaceae* had the lowest relative abundance after S/A supplementation, whereas *Parasuterella* had the lowest relative abundance after the ANTH/FLAV and TPP supplementation (Figure S5).

### 3.2. A Trend Towards Increased Microbiota α Diversity in Older Women Consuming BB

The α diversity of the fecal microbiota in the human trial subjects did not show significant difference across time points for any of the α diversity indices measured (Shannon, Simpson, Chao1, PD, and Observed Species) (Figure S6). The lower sample size, especially of the group of older women, reduced the statistical power of the comparisons. However, investigating the time point-specific distributions of the diversity measures separately for the young and the older women indicated that, for the older group, for most of the measures (with the exception of Shannon) the α diversities at time points W4 and W6 were observed to be similar and higher than that at the pre-intervention W0 time point, indicating an increasing albeit non-significant trend for the elderly (Figure S6). We then investigated this further to check if any differences in the microbiota α diversity were observed by comparing the pre (W0) and post (W4 and W6) intervention time points on a per-individual basis (separately for each age group) (Figure S7a). In the fecal microbiota of five out of six older subjects, the Shannon diversity was increased during the intervention (mean of W4 and W6 aggregated) from the baseline W1 (Figure S7a). Similar results were not observed for the young subjects (Figure S7b).

### 3.3. Distinct CAGs Represented by Health-Promoting Taxa Were Associated with BB Consumption at Each Time Point

Based on β diversity, the fecal microbiota of the older subjects formed a distinct cluster at W4, albeit with high intra-sample variation (Figure S8a). Given the lower sample size and high intra-sample variability, the trends were not significant (PERMANOVA *R*^2^ = 0.03). The fecal microbiota of the younger women showed no β diversity shifts throughout the intervention (PERMANOVA *R*^2^ = 0.002) (Figure S8b).

Despite the lack of clear β diversity in the BB consumption-associated signatures, a significant lower intra-sample microbiota variability at intervention time points was observed for both sub-groups (at W4 versus W2 and W6, and at W6 compared to W2 and W4, respectively) (Figure S8c). This observation could indicate that, in spite of the high inter-individual variability, specific taxa groups may have changed across time points concurrently (enriched or depleted), resulting in a significantly lower inter-individual variability [55].

**Figure 3:**
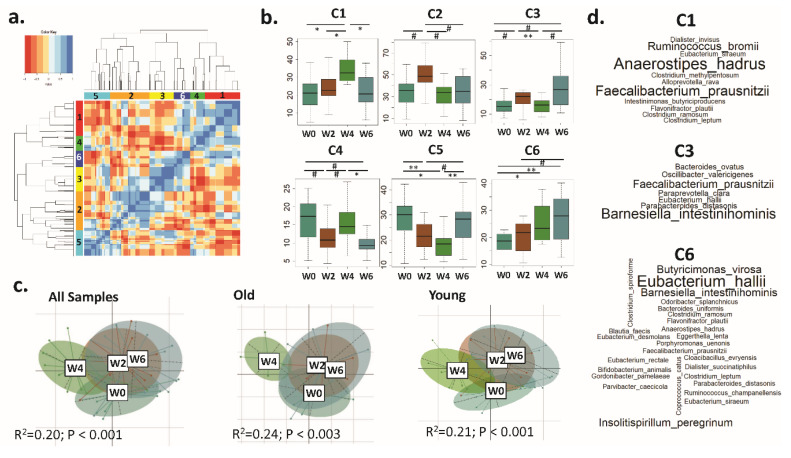
Associations of blueberry (BB) consumption with the enrichment of specific co-abundance taxonomic groups (CAGs). (**a**). Heatmap showing the Kendall tau between the different operational taxonomic units (OTUs) (that is, the OTU to OTU correlations) obtained based on their mean abundances across the different time points. Based on their association patterns, the OTUs were categorized into 6 co-abundance groups or CAGs. The 6 CAGs (C1 to C6) are indicated in colors on the left and top panels. (**b**). Boxplots showing the variation in the OTUs’ cumulated relative abundances (y axis) belonging to the 6 CAGs across the four time points (x axis). *p*_adj_ values showing the significant differences in the CAG abundances (Dunn’s post-hoc test) across time points are indicated: *: *p*_ad_j < 0.05; **: *p*_adj_ < 0.01. Marginal differences are also noted: #: *p*_ad_j < 0.1. (**c**). Principal Coordinates Analysis (PCoA) showing gut microbiota grouping based on the abundances of the 6 different CAGs. The PCoA plots are shown for all the microbiotas aggregated and separated for the old and young sub-groups. The PERMANOVA *R*^2^ and *p* values are indicated in each plot. (**d**). Word clouds showing the species’ enrichment in the CAGs C1, C3, and C6 dominant at either W4 or W6 or both. The species name is proportional to the frequency of that species.

Microbiome configuration analysis offers a more refined approach to monitor microbiota changes compared to individual taxa analysis, because OTUs that co-occur at similar proportions may have trophic and functional interactions relevant for gut ecology [55]. In an analysis of the aggregated microbiota data from all study participants and based on the aforementioned variability trends, six CAGs of OTUs were identified (C1 to C6) (Figure 3a). Interestingly, while the abundance of each of these CAGs exhibited significant differences at W2, W4, and W6 (Figure 3b; Figure S9) when investigating for variability trends in old and young women, no significant differences in the OTUs’ (of the six CAGs) cumulated abundance variation were observed. This indicated that, while the individual constituents may show a high inter-individual variability, the CAGs as a whole exhibit significant time point-specific trends (irrespective of the age group of the participants), even at the individual level (thereby indicating their reliability).

CAG-level PERMANOVA analysis at the different time points revealed significant differences (Figure 3c). The analysis revealed a distinct gut microbiome composition at W4, while W2 and W6 clustered closer; this was observed for both the old and young sub-groups. No significant differences in the cumulated abundances variation in the OTUs belonging to the six CAGs separately within the old and the young were observed (Figure S9).

The C1, C3, and C6 CAGs’ cumulated abundances increased significantly with BB consumption (at W4 and W6). C1 increased from W0 to W4 and decreased at W6, whereas C3 and C6 progressively increased from W0 to W6 (Figure 3b). Several health-relevant species were abundant in these CAGs—e.g., *Faecalibacterium prausnitzii*, *Barnesiella intestinihominis*, *Eubacterium halii*, *Anaerostipes hadrus*, and *Ruminococcus bromii* (Figure 3d). These results indicate the putative beneficial effect of BB consumption on the gut microbiota.

### 3.4. Antioxidant Activity (FRAP) Is Significantly Associated with the Faecal Microbiota

The levels of plasma CRP, glucose, and FRAP assay measures were collected at W0 and W6 for 10 young and 5 old women. Using PERMANOVA analysis, the association of the gut microbiota at both OTU and CAG level with CRP, glucose, and FRAP was investigated (Table 2). No association between the gut microbiota composition with either the CRP or glucose levels was observed. However, the FRAP assay measures showed significant association with the gut microbiota at both the OTU and CAG level (Table 2).

**Figure 4:**
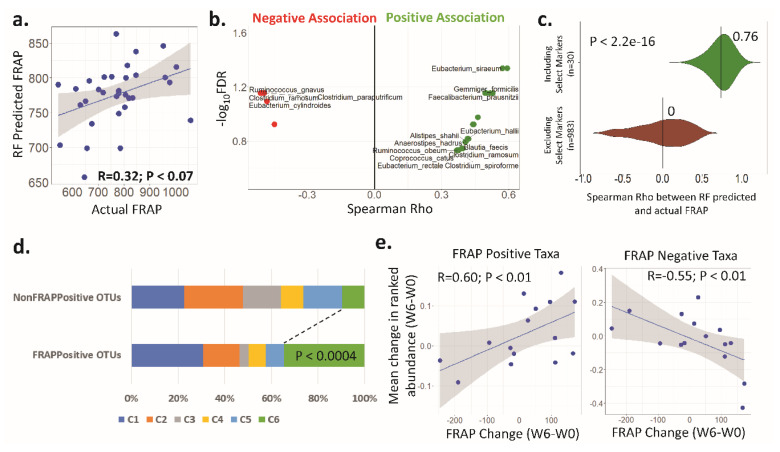
Fecal microbiota components were associated with increased ferric-reducing antioxidant power (FRAP) measures and with the co-abundance groups (CAGs) enriched upon blueberry (BB) consumption. (**a**). Scatter plot showing the correlation between the actual and the Random Forest-predicted FRAP values. (**b**). Violin plot showing the association of the top 30 operational taxonomic unit (OTU) markers with the FRAP assay measures. X axis: Spearman Rho between the OTU abundances and the FRAP assay measures. Y axis: log of the Benjamini-Hochberg (BH) false discovery rate (FDR) with base 10. OTUs on the left: negatively associated; OTUs on the right: positively associated. Green color: the top 30 OTU markers showing significant association with FRAP measures (with FDR < 0.2) (positively associated); red color: negatively associated markers. (**c**). Bean plots showing the Spearman Rho measures distribution obtained for the predicted and the actual FRAP across the 100 iterations of the two variants of Random Forest models. (**d**). Stacked bar plots showing the relative representation of the different CAGs in the FRAP positive OTUs and the other non-marker OTUs. Seventeen out of the 25 FRAP-positive OTUs belonged to either CAG C1 (8) or C6 (9). Fishers’ exact test showed a significant association between C6 and the FRAP-positive OTUs (indicated in the Figure). (**e**). Scatter plots showing the correlation between the FRAP-positive and FRAP-negative OTUs’ mean abundances change across time points with the corresponding changes in FRAP measures.

Random Forest models were built to predict the FRAP assay measure of an individual (at a given time point) based on their gut microbiota composition (at the OTU level) (as described in Materials and Methods). The validation of these models using an iterative leave-one-out-strategy (i.e., excluding from the training model the sample to be predicted) indicated a marginally positive Spearman correlation of 0.32 (*p* < 0.07), further indicating an association of the microbiome with plasma antioxidant activity (Figure 4a). Thus, in the current study the OTUs were initially ranked in increasing order of their feature importance scores, and subsequently the variation in these feature importance scores across them was investigated.

An exponential increase in scores for the last 150 taxa (or OTUs) as compared to the rest was observed (Figure S10a). The list of the 150 taxa was filtered by selecting only those OTUs that showed significant association with FRAP measures with BH-corrected FDR < 0.1 (Figure S10b). This provided the 30 top predictors of FRAP measures at the OTU level (Figure 4b). While 25 of these top markers were positively associated with FRAP (FRAP-positive markers), five were negatively associated with FRAP (FRAP-negative markers).

The efficacy of these top 30 markers was further evaluated using two variants of iterative Random Forest models, one using only these top 30 and the other using the remaining OTUs (Table S1). A comparison of the performances of the two variants indicated that models created using only these 30 top markers could still predict FRAP measures with a median Spearman Rho of 0.76 (*p* < 1 × 10^−5^), which was significantly higher than those created using the remaining 983 non-marker OTUs (median Rho = 0.02) (*p* < 2.2 × 10^−16^) (Figure 4c).

Distinct changes in the markers of FRAP assay measures during the intervention time points were as follows. The FRAP assay measures increased for 9 of the 15 subjects (4 out of 5 old, 5 out of 10 young) (Figure S10c). An overlap between some of the species that were positively associated with FRAP measures and those enriched during BB consumption was observed (Figure 4b). These included gut bacterial species such as *F. prausnitzii*, *E. halii*, *E. siraeum*, *C. catus*, and *A. hadrus*.

The representation of the different previously identified CAGs in the subset of FRAP-positive markers was explored. Seventeen out of the 25 FRAP positive markers belonged to either the C1 or the C6 CAGs that were significantly enriched in W4 and W6 time points, respectively (Figure 3b; Figure 4b) Thus, a subset of taxa that were identified as belonging to reportedly beneficial microbial groups enriched in the later stages of BB consumption also showed positive associations with the antioxidant activity. This indicates that BB consumption is associated with microbiome changes that are positively associated with antioxidant activity.

Interestingly, the across-time point changes in FRAP assay measures showed negative associations with the corresponding changes in the plasma glucose levels (Spearman Rho = −0.46; *p* < 0.05) (Figure S10d). A similar negative association was also observed between the FRAP positive-markers and the plasma glucose levels. Thus, these results overall seem to suggest a step-wise association between BB consumption and plasma glucose levels, wherein the consumption of BBs is associated with the enrichment of specific taxonomic groups, and a subset is positively associated with circulating antioxidant activity, which in turn is negatively associated with the plasma glucose levels. The associations of these OTUs with FRAP were both sample-specific and distinct for time points.

## 4. Discussion

We have previously reported the reduction in the fecal microbiota α diversity due to the loss of fastidious taxa while the microbiota is adapting to the in vitro conditions of the artificial colon model [39,56]. To retain much of the stool diversity throughout the fermentation period, the basal fermentation medium was supplemented with a mix of indigestible and prebiotic carbohydrates often used in continuous in vitro systems [57]. Importantly, the supplementation of the fermentation medium with the polyphenol-rich fractions ANTH/FLAV, PACs, or prebiotic MIX substrates resulted in a favorable (i.e., health-associated) microbiota profile. Although the content of these fractions was not yet investigated by chromatographic separation, the fractions tested were prepared in the same way as in multiple previous publications, and so they are directly comparable in terms of evaluating their bioactive properties. The α diversity and the abundance of the major microbiota families *Lachnospiraceae* and *Bacteroidaceae* were comparatively higher, whereas the Enterobacteriaceae relative abundance was lower compared to TPP and S/A supplementation. The sugar content of the S/A fractions and potential residual sugars in the TPP fraction, which would be expected to be absorbed in the small intestine in humans, may have resulted in the significantly increased relative abundance of these organisms that are potent utilizers of simple sugars [58]. Apart from this explanation, *Enterobacteriaceae* have been reported to be involved in the metabolism of polyphenols in the gut [27,59]. Importantly, not all *Enterobacteriaceae* are harmful, with some playing an important role in the “healthy” gut microbiota [9].

Bifidobacteria residing in the colon may be utilizing the polyphenolic sugar content that reaches distal gastrointestinal parts [60,61]. TTP followed by PACs (but not ANTH/FLAV) supplementation were the most efficient additives to maintain the *Bifidobacterium* abundance levels in the suboptimal in vitro environment. Previous in vitro fermentation studies have yielded conflicting results on the effect of polyphenol-rich fractions on *Bifidobacterium* spp. abundance in the microbiota [21,62]. Limitations of the in vitro systems and baseline microbiota variations may have contributed to these discrepancies. Human studies have confirmed some effect of polyphenols on bifidobacteria [63].

We observed a trend for microbiota α diversity increase in the group of older women consuming BB, and although the β diversity did not change throughout the intervention period, health-relevant taxa were significantly enriched with BB consumption in subjects of both age groups. We acknowledge the limitations of the small sample size in this study, due in part to the complexity of running a human dietary intervention trial in which the primary objective was to test the effect of BB consumption on the human muscle progenitor cell (hMPC) function [64]. Nevertheless, the current study serves adequately as a pilot study to investigate the potential of regular BB consumption to improve the microbiota diversity in older healthy people. Maintaining microbiota diversity is relevant throughout the lifespan. Risk factors for non-communicable disease are associated with a Western lifestyle [65,66,67]. Decreased gut microbiota diversity as in low species richness and low counts of bacterial genes may correlate to metabolic disease, and therefore global microbiota modulations can promote health in the general population [68,69].

The enrichment of the fecal microbiota in *Anaerostipes hadrus, F. prausnitzii*, and to a lesser extent *Ruminococcus bromii* (CAG C1)—all taxa of the “healthy” microbiota [70]—two weeks after BB intervention indicated a potential microbiota adaptation to BB consumption. Enrichment in the major fibrolytic taxon *R*. *bromii* [71,72] may represent an adaptation to the regular fibre derived from whole BB fruit. *Ruminococcus bromii* releases substrates from complex polysaccharides that other microbiota members such as *A*. *hadrus* and *F. prausnitzii* can metabolize [73]. *Anaerostipes hadrus* is a butyrate producer previously reported to be stimulated by prebiotic fibres [74,75]. The ecological context is important when evaluating the health benefit of taxa that are “prebiotically” stimulated. In the case of *A*. *hadrus*, it was reported that it exerted beneficial outcomes in “healthy” microbiota and adverse in dysbiotic microbiota in a mouse model [76], potentially involved in energy harvesting and blood glucose [77]. *Faecalibacterium prausnitzii* is a key butyrate producer with anti-inflammatory properties, and its reduced abundance in the microbiota has been associated with various gastrointestinal conditions [9,78]. A few studies in mice and humans have shown *Faecalibacterium* responsiveness to polyphenols, accompanied by metabolism improvement [79,80,81].

Taxa such as *E*. *hallii*, *B*. *intestinihominis*, and *Butyrisimonas virosa* (CAG C6) and *B*. *intestinihominis* and *F. prausnitzii* (CAG C3) showed a gradual increase in abundance from baseline towards later intervention time points. The increased abundance of *E*. *hallii*, a butyrate producer of the Lachnospiraceae family [70], identified here to be associated with BB consumption, may contribute to improved insulin sensitivity according to in vivo and human studies [82,83]. Other *Eubacterium* spp. taxa of the Lachnospiraceae family (e.g., *E. ramulus* and *E. rectale*) may be involved in the metabolism of polyphenolic compounds (e.g., flavonols, flavanols, and lignans) [84,85]. Similarly, intervention with a polyphenol-rich diet was associated with enrichment in *B. intestinihominis* and improved metabolism in mice and humans [86,87].

There was some moderate albeit significant correlation of OTUs and CAGs with the FRAP measurements that, in turn, positively correlated with BB consumption, especially in the older group. Conversely, FRAP measurements were negatively correlated with plasma glucose. Many studies on healthy adults have contributed evidence for the antioxidant and anti-inflammatory benefits of the regular consumption of polyphenol-rich foods, such as BB and other berry fruits [88]. There is evidence from cohort and clinical studies of reduced all-cause mortality, lower risk of CVD, improved insulin sensitivity, and lower type 2 diabetes (T2D) risk associated with BB and specifically anthocyanin intake [32]. Importantly, in older age groups anthocyanins appear to lower the risk of cognitive decline [32]. However, there is a lack of human studies investigating the consumption of polyphenol-rich berries, metabolic improvement, and the microbiota, with the majority of relevant data derived from animal studies [27,89,90,91,92]. Importantly, we recognize critics of FRAP assays and that, according to the literature, more oxidative damage markers should be added to allow robust conclusions to be drawn [88].

Here, we report taxa that not only were associated with BB consumption forming distinct CAGs (i.e., *E. hallii*, *B*. *intestinihominis*, *A*. *hadrus, F.prausnitzii*) as discussed, but that taxa that mostly belong to the significant CAGs were associated with improved FRAP measurements. In this part of the analysis, we found that *E*. *siraeum* (*Clostridium* cluster IV Ruminococcaceae taxon) and the phylogenetically close *F. prausnitzii* and *G. formicilis* [70] were positively associated with FRAP. Interestingly, in humans serum markers of insulin resistance were associated with reduced *E*. *siraeum* and *Butyrivibrio crossotus* abundance [93]. Conversely, *G. formicilis* and *C*. *catus*, identified in this study to be positively associated with FRAP, were associated with obesity [94,95,96,97].

## 5. Conclusions

In vitro conditions place constraints on microbiota responsiveness to supplementation tests [98]. Notwithstanding this, the investigational studies as presented here offer a straightforward experimental model to test the initial hypothesis and provide insight into informed in vivo study design [99]. In future studies, the potential of the in vitro-identified microbiota response to BB polyphenol-rich fractions can be extended to the development of next-generation symbiotics.

The human study contributed evidence for specific microbiota modulation due to BB consumption in correlation with antioxidant activity in healthy adults. The association of fibrolytic taxa with whole BB consumption may indicate that the BB can contribute to health by both its polyphenolic content and its fibre content that, in effect, may render the fibre-bound polyphenols more accessible to microbiota fermentation [100]. In the context of healthy ageing, BB consumption may increase colonic short chain fatty acid (SCFA) production through fiber contribution to the fibrolytic members of the microbiota and promote health [101]. Importantly, non-pathobionts in the “healthy” microbiota, such as the taxa *C*. *catus* or *A*. *hadrus* mentioned in our study, may play a variant role within a different health context and in response to external dietary stimuli [102,103]. Strain-level identification is important in order to explain why individuals may respond differently to microbiota modulation [101,104]. Interindividual variation can be of relevance in the way dietary polyphenols impact health, given the fact that their bioavailability largely depends on the gut microbiota enzymatic armor [105]. Future large-scale clinical studies including both women and men and examining the metabolic impact of BB consumption in correlation with microbiota changes, inflammatory markers, and gender will allow for a deeper understanding of the role of BB consumption in human health. At the same time, a detailed chromatographic analysis of the BB fractions described here is desirable to generate greater granularity and detail on what individual compounds are present in each starting fraction, and what they are metabolized into, in the context of the microbiome changes described already in this report.

## Figures and Tables

**Table 1:** Human study participant demographics.

	Young (*n* = 10 ^a^)	Old (*n* = 6)
Age (yrs)	28 ± 2	69 ± 2
Weight (kg)	64.31 ± 2.33	62.44 ± 3.84
Height (cm)	166.3 ± 1.5	161.3 ± 2.9
BMI (kg/m^2^)	23.3 ± 0.9	24.2 ± 2.0
Glucose (mg/dL, Range: 74–106)	89.5 ± 2.44	98.0 ± 1.7
CRP (mg/L, Range: <1.1)	1.21 ± 0.33	0.76 ± 0.28

All values are presented as means ± standard error. BMI: body mass index; CRP: C-reactive protein. ^a^ One outlier value excluded.

**Table 2:** Gut microbiota composition was significantly associated with ferric-reducing antioxidant power (FRAP). *R*^2^ and *p* values of the PERMANOVA analysis associating the clinical parameters with the gut microbiota at the operational taxonomic unit (OTU) and co-abundance group (CAG) level are shown in the table.

Clinical Indicator	OTU-Level Microbiota		CAG-Level Microbiota	
	*R* ^2^	*p* Value	*R* ^2^	*p* Value
CRP	0.03	0.95	0.02	0.81
Glucose	0.03	0.54	0.02	0.79
FRAP	0.04	0.01	0.06	0.05

## Supplementary Materials

The following are available online at https://www.mdpi.com/2072-6643/12/9/2800/s1: Figure S1: Boxplots showing the supplementation-independent significant decrease in α diversity measures (a. Shannon and b. Observed Species) for 24 h fermentation. Figure S2: Observed Species α diversity of the fecal microbiota after different BB polyphenol-rich fractions supplementations at 16 h and 24 h. Figure S3: Principal Component Analysis (PCoA) based on Weighted Unifrac distances of microbiota after in vitro fermentation with BB polyphenols. Figure S4: Fecal microbiota compositional description at Family level during 24 h in vitro fermentation with BB polyphenol-rich fractions. Figure S5: Differentially abundant taxa (genus level) in the fecal microbiota after in vitro supplementation with BB polyphenol-rich fractions (average relative abundance < 1%). Figure S6: Alpha diversity of the fecal microbiota of a. young and b. older women of the human trial across all time points. Figure S7: Shannon diversity showing the fecal microbiota α diversity development pre (W0) and post (mean of W4 and W6) BB consumption intervention. Figure S8 Variation in within cohort beta diversity for the Young and Old women. Figure S9: Variation in the cumulated abundances of the OTUs belonging to the six CAGs specifying for old and the young women. Figure S10: Identification of FRAP responsive taxa, their variation across time points and their association with glucose levels. Table S1: *p*_adj_ values of Wilcoxon Signed Rank tests comparing the abundances of the various taxa in fermenter samples belonging to the various supplementation groups. Table S2: Taxonomic classifications of (A) FRAP-positive and (B) FRAP-negative OUT markers obtained using SPINGO.
