# Supplementary material for: Whole Blueberry and Isolated Polyphenol-Rich Fractions Modulate Specific Gut Microbes in an In Vitro Colon Model and in a Pilot Study in Human Consumers
Source: Nutrients. 2020 Sep 12;12(9):2800. doi: 10.3390/nu12092800 (PMC7551244; doi:10.3390/nu12092800)
Supplement: Supplementary file 1 [file nutrients-12-02800-s001.pdf]

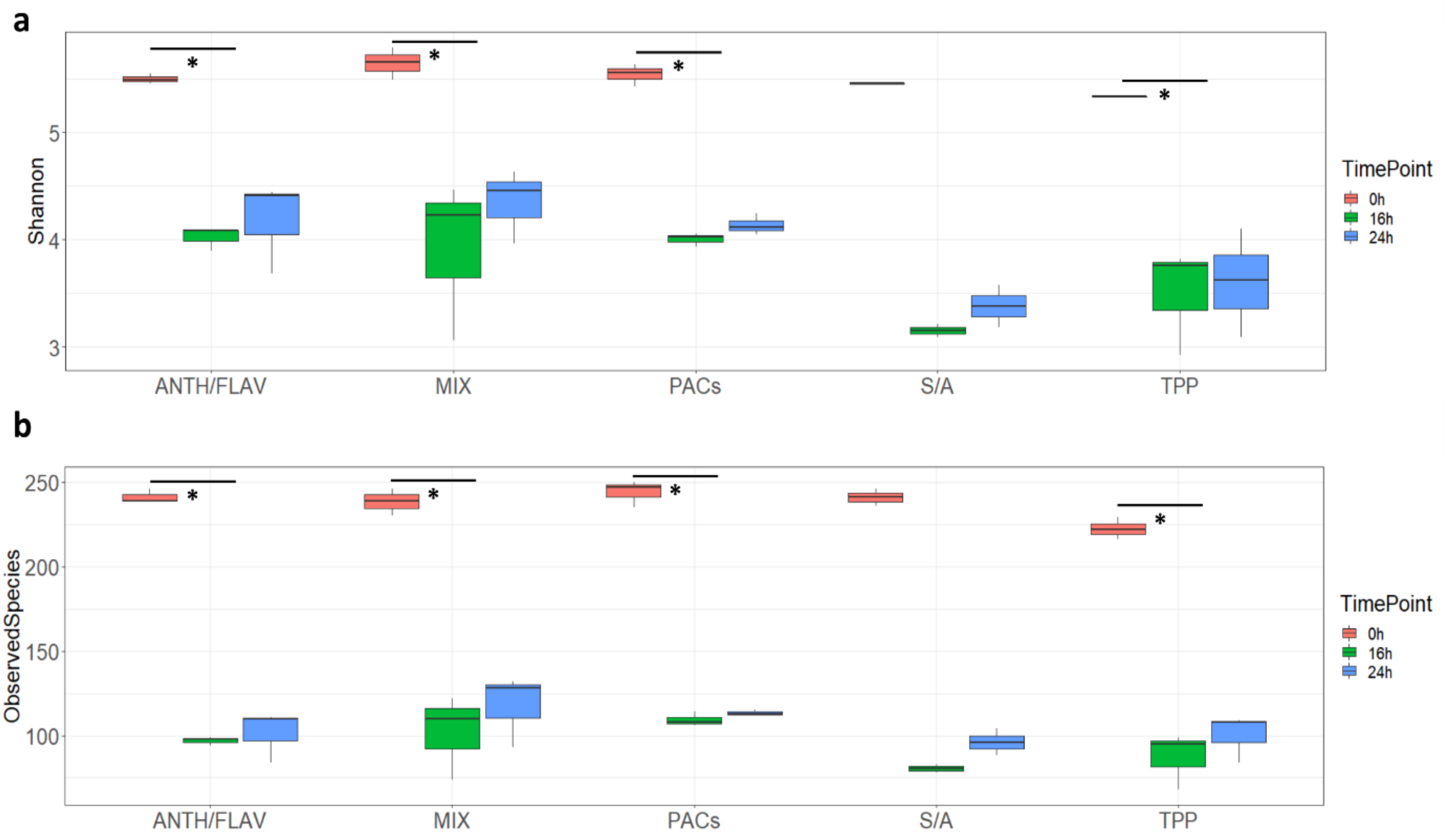

Figure S 1 Boxplots showing the supplementation-independent significant decrease in α diversity measures (a. Shannon and b. Observed Species) during the 24 h of fermentation. Time points: 0 h (baseline; in pink colour); 16 h (in green) and 24h (in blue). Significant differences for comparisons combining 16 h and 24 h data are indicated on bar plots: * $p_{adj}<0.05$ (post-hoc Dunn's test with BH p_{adj}). ANTH/FLAV: anthocyanin/flavonol glycoside supplementation; MIX: prebiotic fibres mix supplementation; PACs: proanthocyanidin supplementation; S/A: sugar/acid fraction supplementation; TPP: total BB polyphenols.

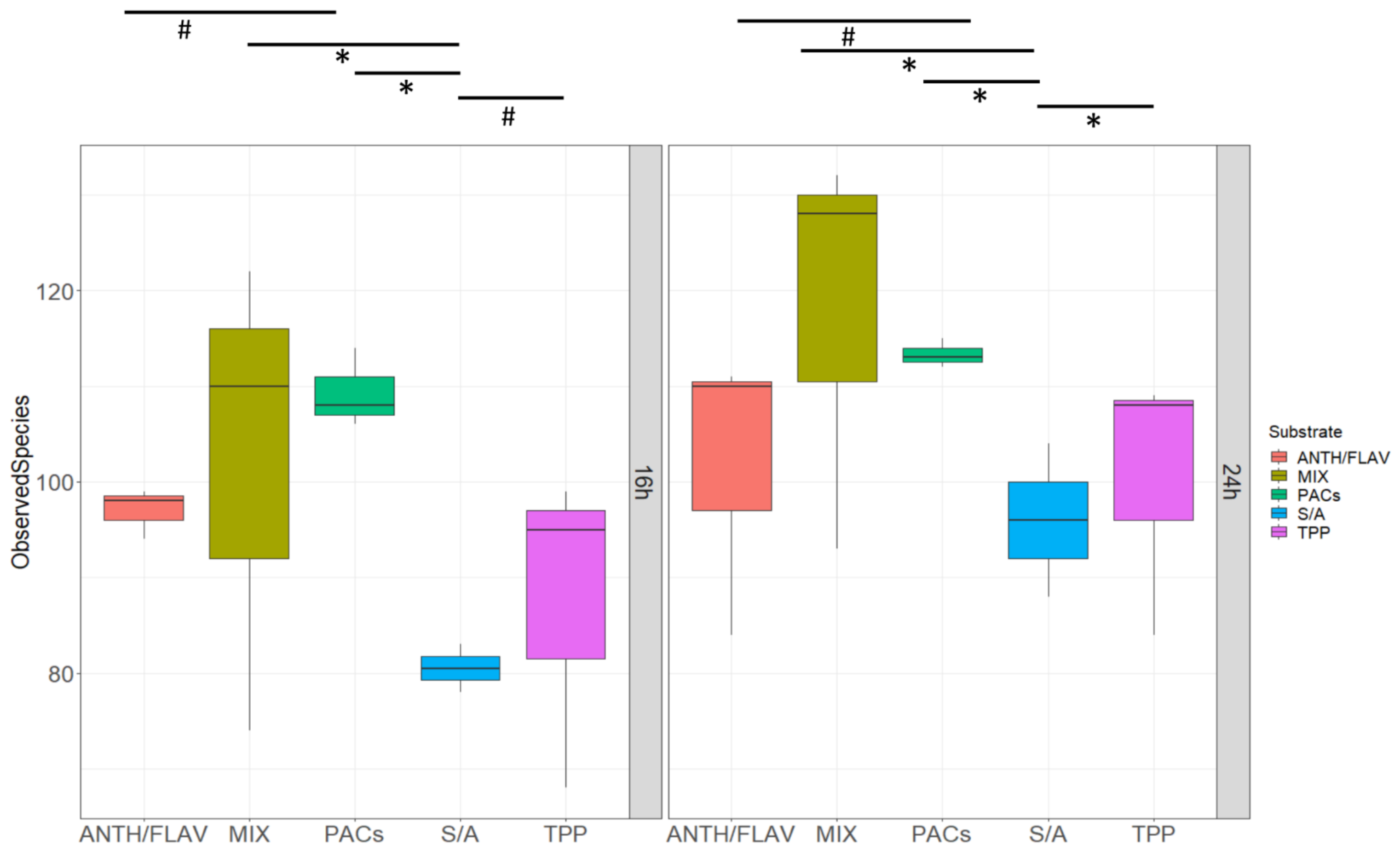

Figure S 2 Observed Species α diversity of the faecal microbiota after different BB polyphenol-rich fractions supplementations at 16 h and 24 h. Significant differences (post-hoc Dunn's test with BH p_{adj}) across supplementations are indicated in bar plots: * $p_{adj}<0.05$. Marginal differences are also indicated: # $p_{adj}<0.10$. ANTH/FLAV: anthocyanin/flavonol glycoside supplementation; MIX: prebiotic fibres mix supplementation; PACs: proanthocyanidin supplementation; S/A: sugar/acid fraction supplementation; TPP: total BB polyphenols.

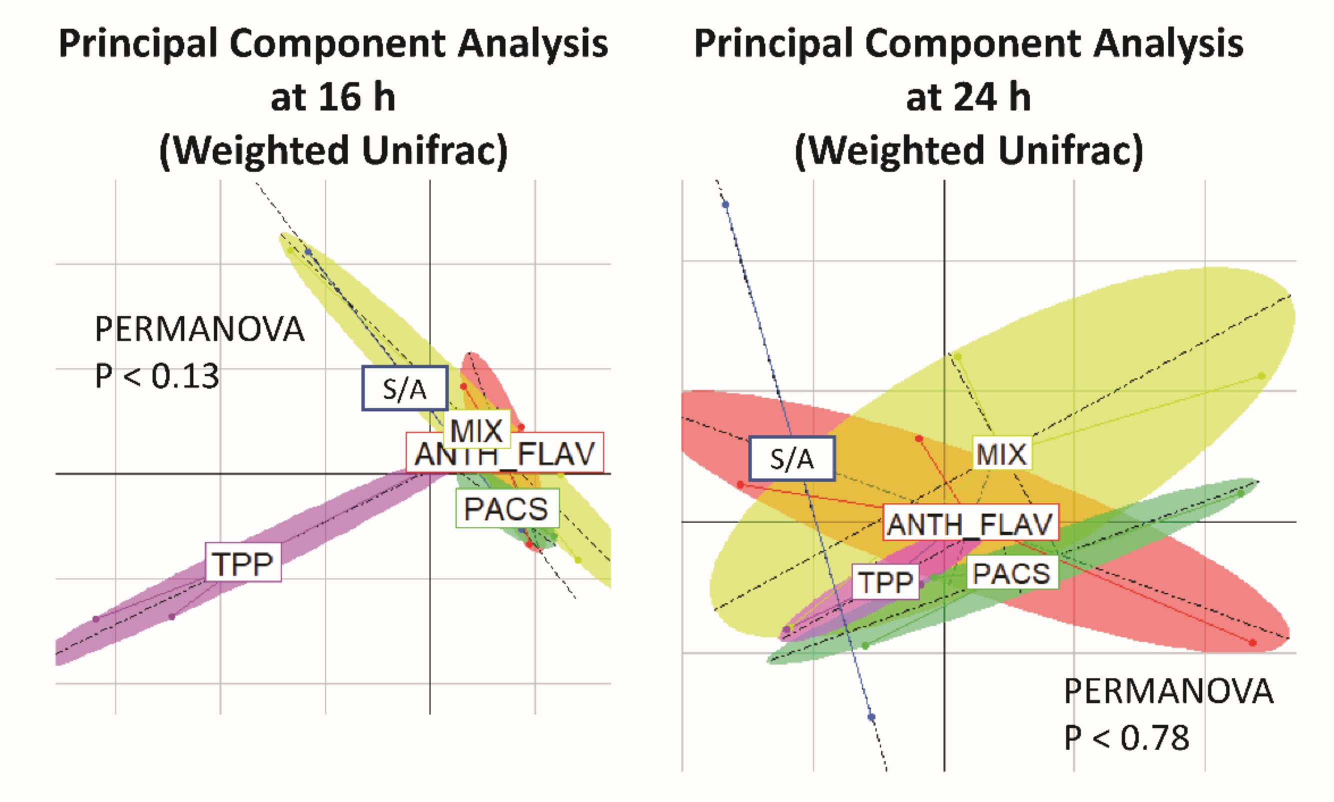

Figure S 3 Principal Component Analysis (PCoA) based on Weighted Unifrac distances of microbiota after *in vitro* fermentation with BB polyphenol-rich fractions at 16 h and 24 h. PERMANOVA p values are indicated. ANTH/FLAV: anthocyanin/flavonol glycoside supplementation; MIX: prebiotic fibres mix supplementation; PACs: proanthocyanidin supplementation; S/A: sugar/acid fraction supplementation; TPP: total BB polyphenols.

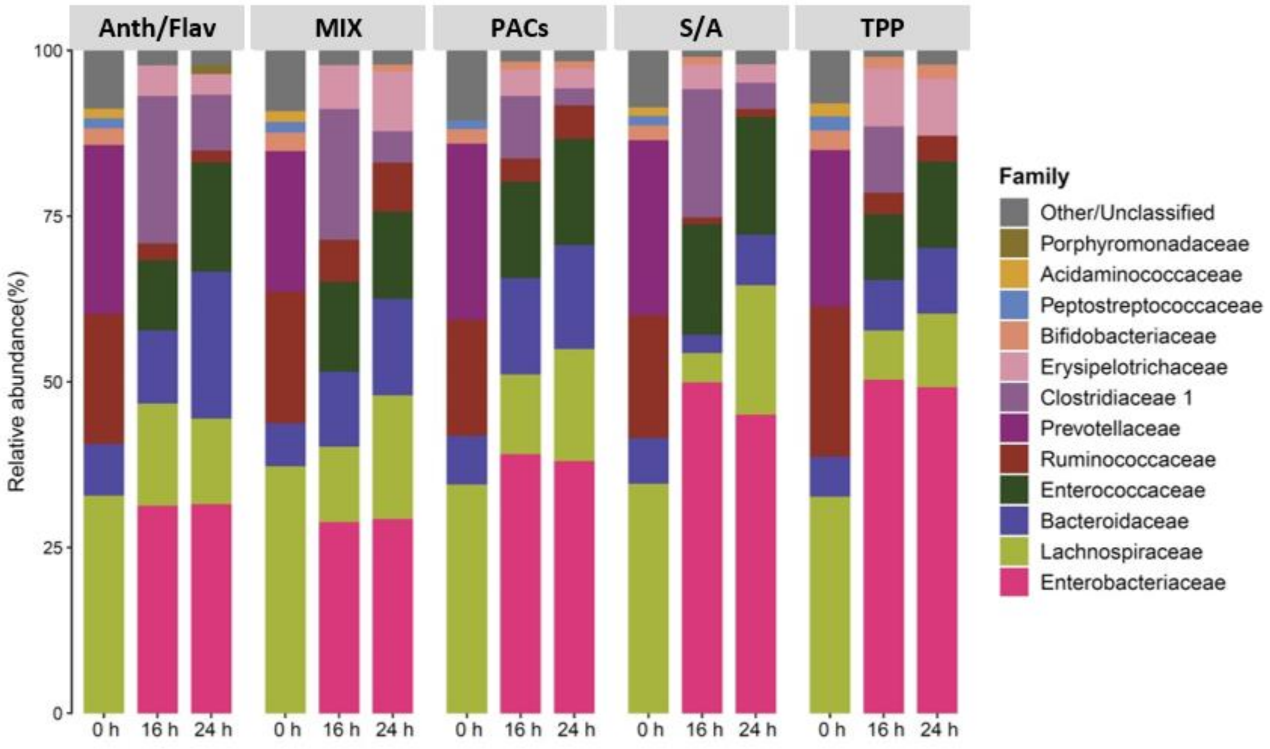

Figure S 4 Faecal microbiota compositional description at Family level during 24 h *in vitro* fermentation with BB polyphenol-rich fractions. Bacterial families present at >1% relative abundance are presented. Time points: 0 h (baseline); 16 h; 24 h. ANTH/FLAV: anthocyanin/flavonol glycoside supplementation; MIX: prebiotic fibres mix supplementation; PACs: proanthocyanidin supplementation; S/A: sugar/acid fraction supplementation; TPP: total BB polyphenols.

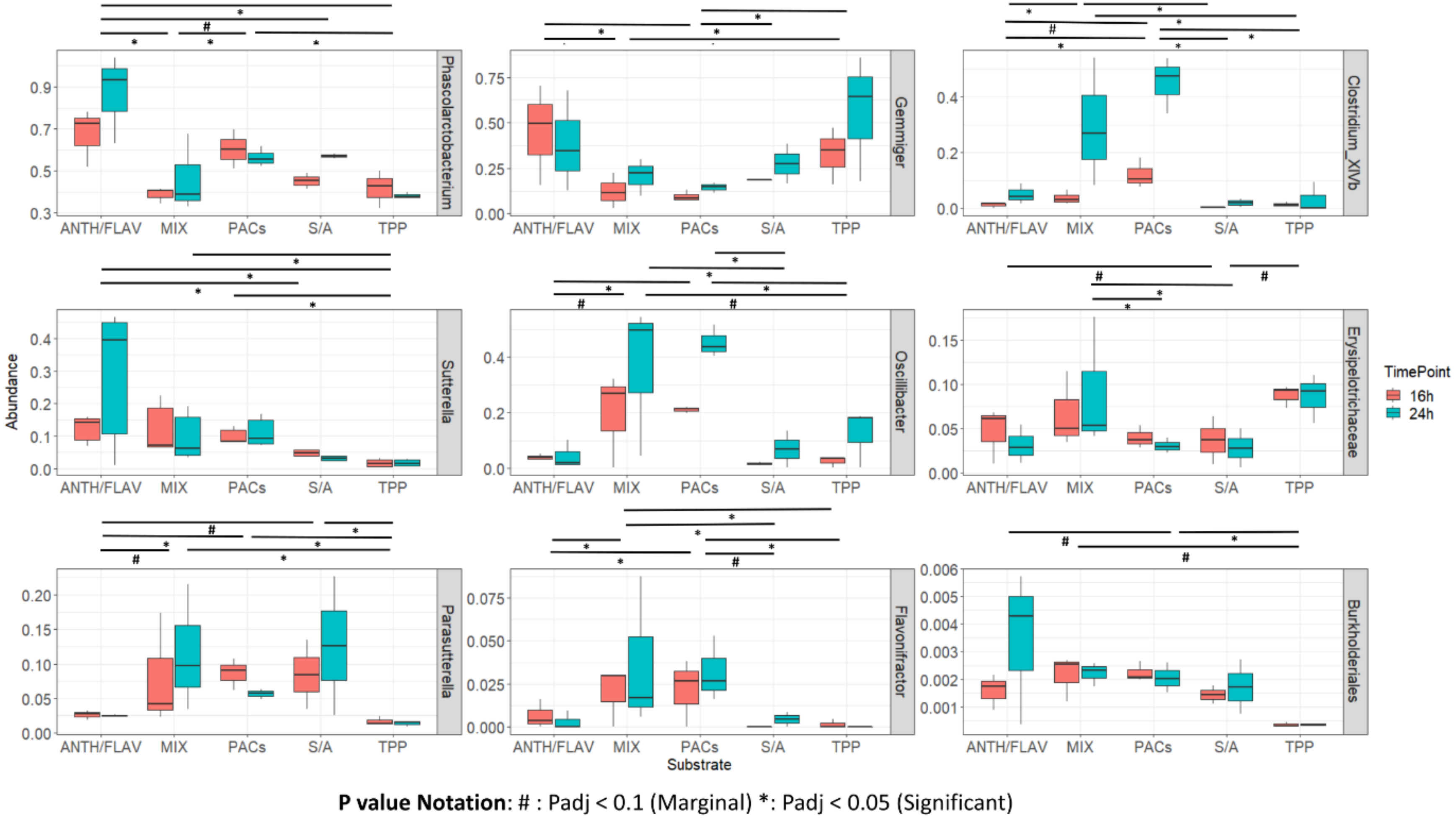

Figure S 5 Differentially abundant taxa (genus level) in the faecal microbiota after *in vitro* supplementation with BB polyphenol-rich fractions during the 24 h fermentations (average relative abundance of taxa <1%). The results of relative abundance from 16 h and 24 h fermentations are shown in pink and green colour, respectively. Significant differences for comparisons combining 16 h and 24 h data (post-hoc Dunn's test with BH p_{adj}) across supplementations are indicated in bar plots: * $p_{adj}<0.05$. Marginal differences are also indicated: # $p_{adj}<0.10$. ANTH/FLAV: anthocyanin/flavonol glycoside supplementation; MIX: prebiotic fibres mix supplementation; PACs: proanthocyanidin supplementation; S/A: sugar/acid fraction supplementation; TPP: total blueberry polyphenols.

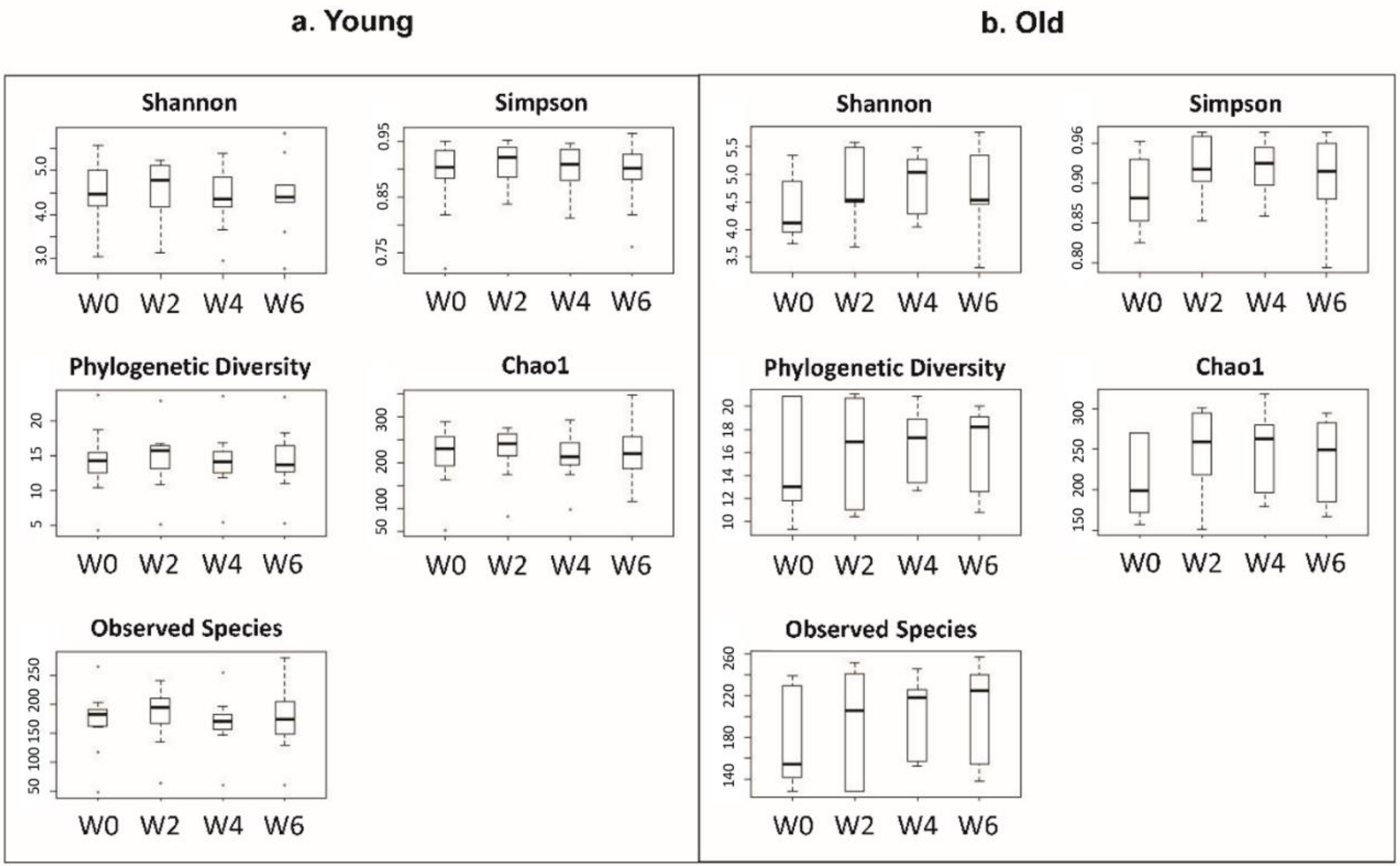

Figure S 6 Alpha diversity of the faecal microbiota of **a.** young and **b.** older women of the human trial across all time points. Time points: W0, W2, W4 and W6. Alpha diversity measurements: Shannon index, Simpson index, Phylogenetic Diversity (PD), Chao1, Observed Species.

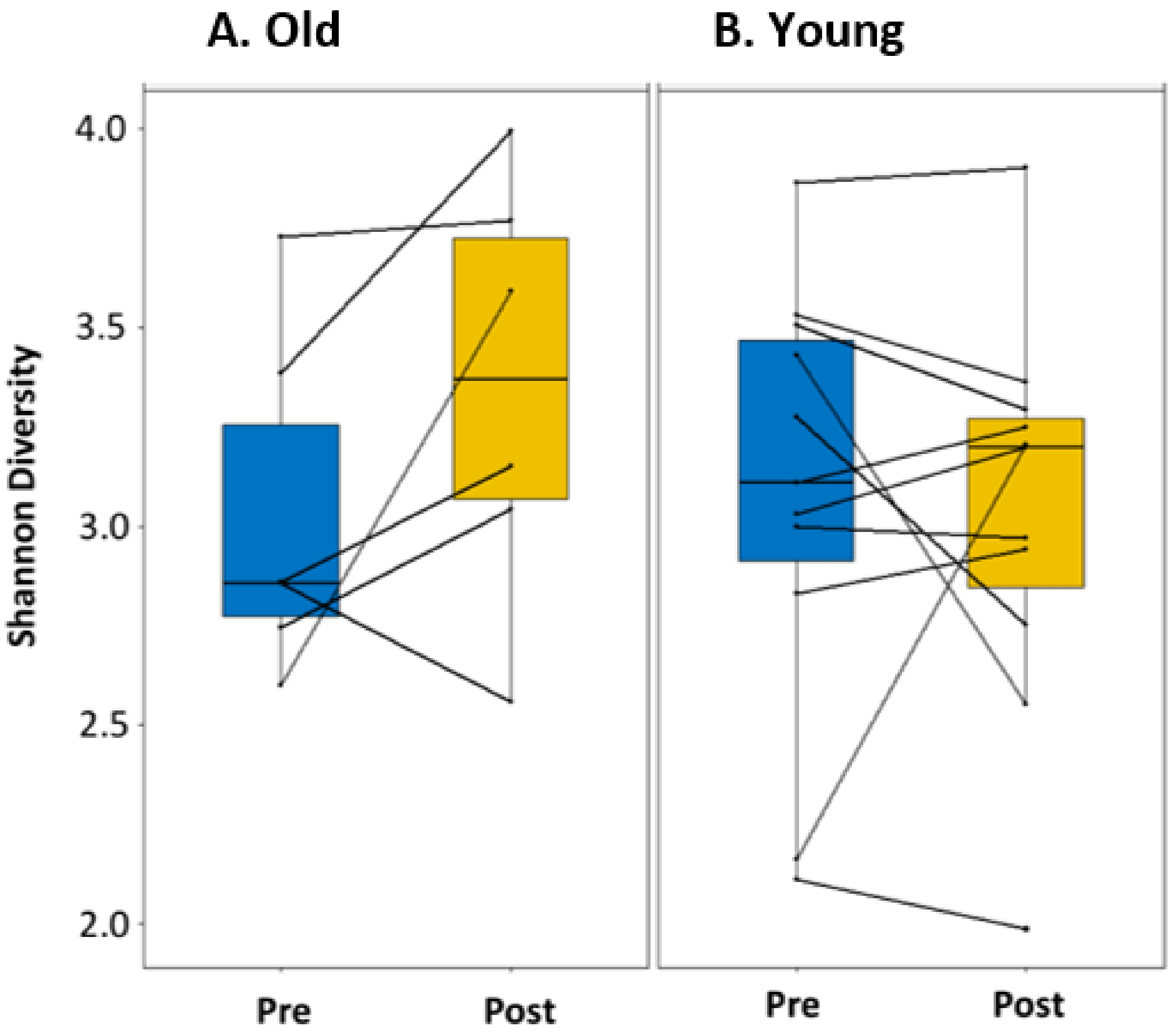

Figure S 7 Shannon diversity showing the faecal microbiota α diversity development pre (W0) and post (mean of W4 and W6) BB consumption intervention. The consumers were separated in **a.** old and **b.** young women. The lines indicate the diversity transition of each participant.

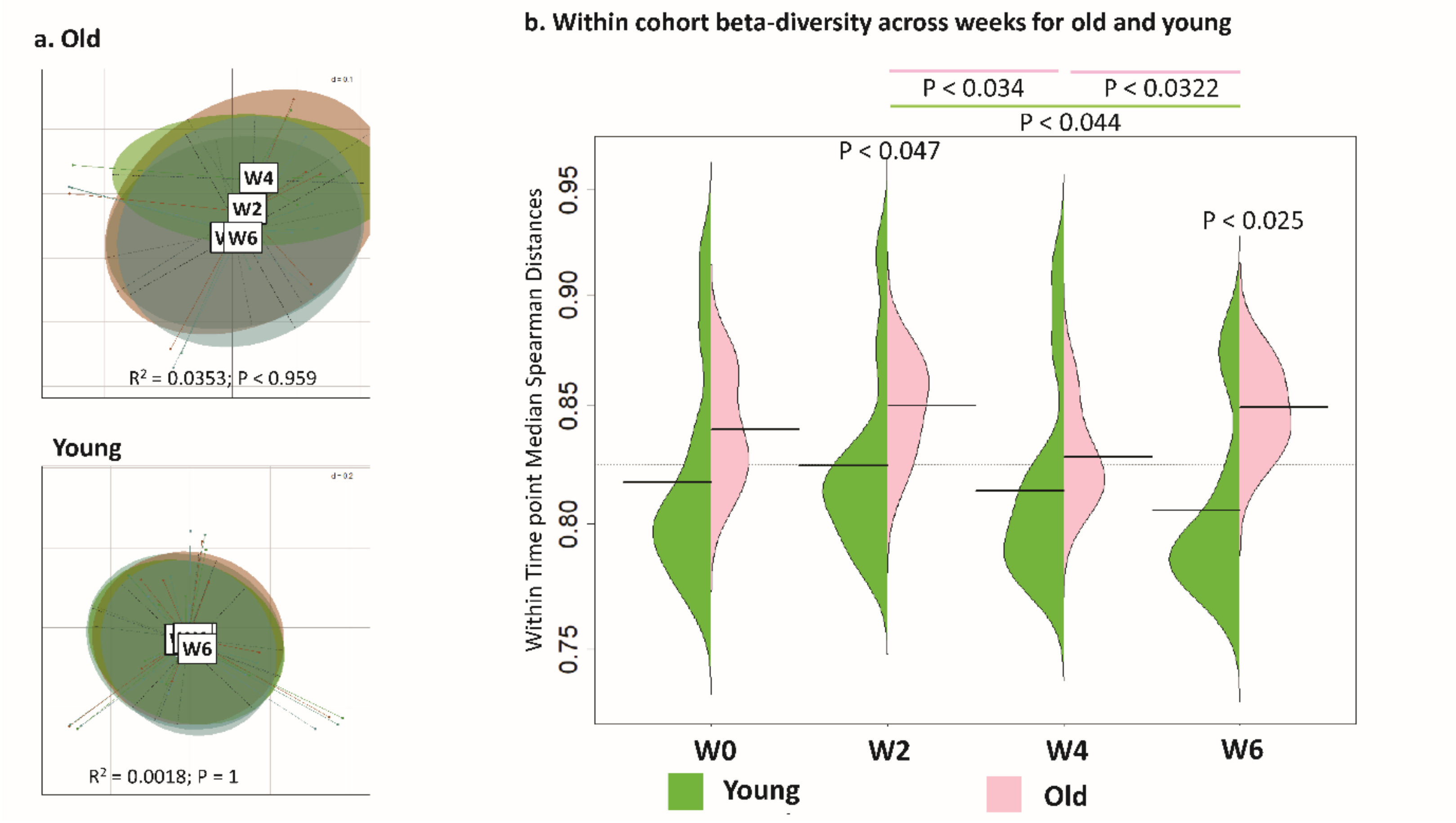

Figure S 8 Variation of within cohort beta diversity for the Young and Old women **a.** Principal component analysis (PCoA) based on Spearman distances showing the faecal microbiota grouping during the intervention time points for young and old women. W0, W2, W4, W6: faecal sample collection time points. The R^2 and p value of the PERMANOVA analysis of each sub-cohort is indicated. **b.** Beanplots showing the intra-time point variations of the faecal microbiota composition of the subjects belonging to the young (green) and old (pink) age groups (green). Significant differences of these variations across time points and sub-groups (computed using Mann-Whitney tests as described in the Materials and Methods section) are indicated as follows. While p values indicated below horizontal green bars denote the significant differences between the corresponding time points for the young age group, those indicated below horizontal pink bars indicate the same for the older age group. The p values indicated on top of each beanplot indicate significant differences between the older and young women for the same time point.

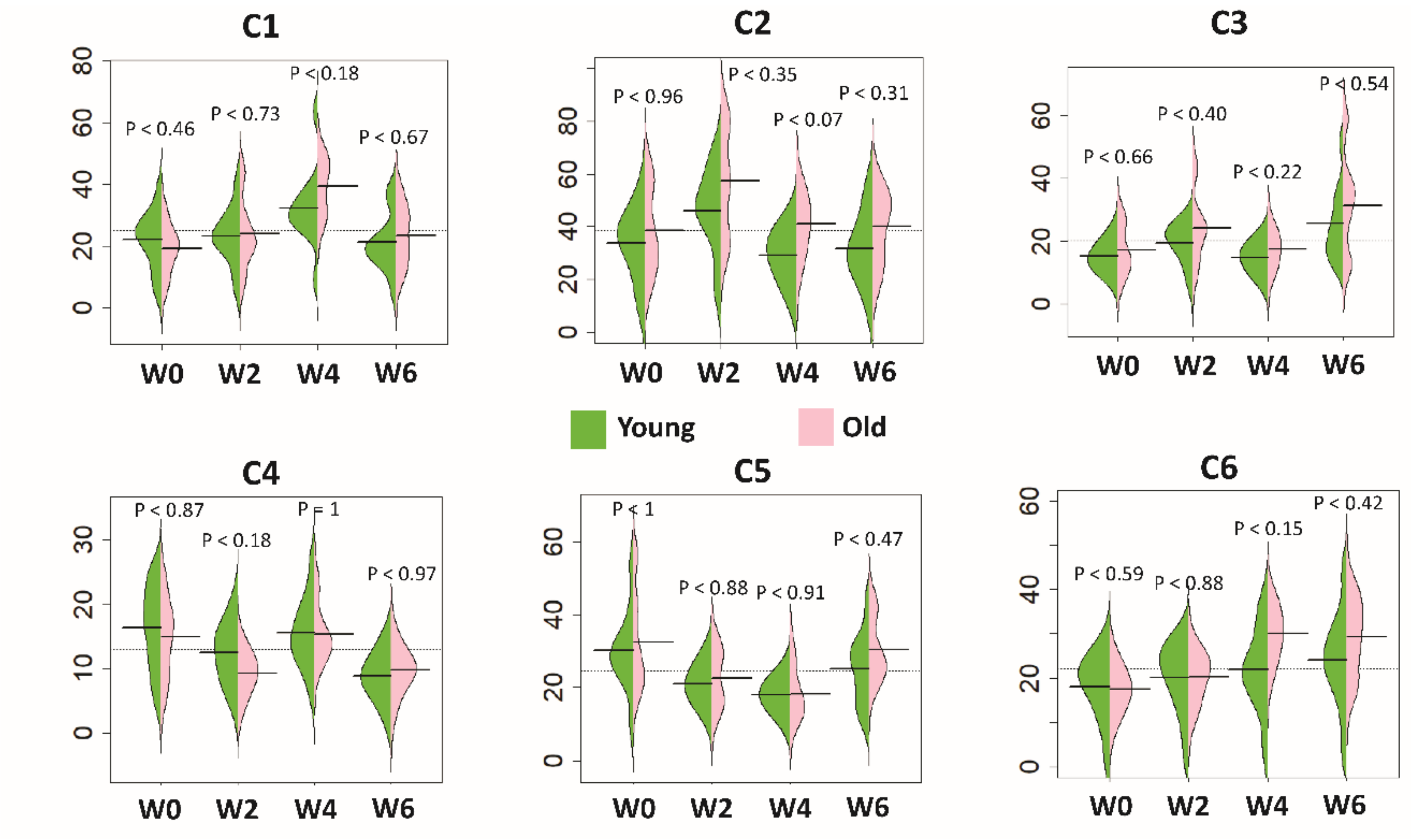

Figure S 9 Variation of the cumulated abundances of the OTUs belonging to the six CAGs specifying for old and the young women. X axis: the intervention time points; y axis: % average relative abundance.

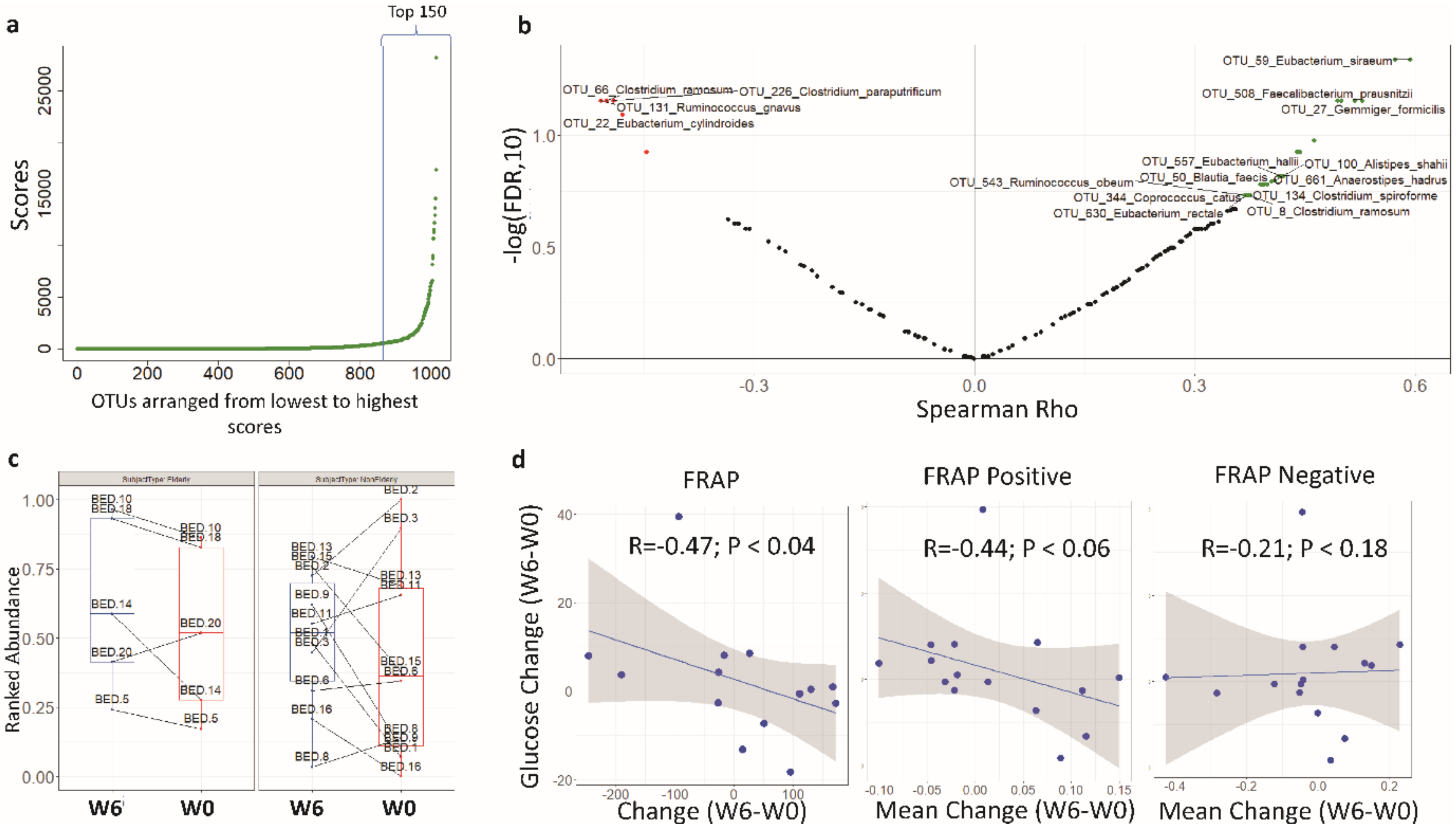

Figure S 10 Identification of FRAP responsive taxa, their variation across time points and their association with glucose levels. **a.** Variation of Feature Importance Scores (Mean Decrease in GINI) of all OTUs. The OTUs are arranged in increasing order of their feature importance scores. The sharp increase of feature importance scores for the top 150 OTUs is shown. **b.** Violin plot showing the association of the 150 OTUs with the highest FRAP measures. X axis: Spearman Rho between the OTU abundance and the FRAP assay measures. Y axis: -log of the BH FDR with base 10. OTUs on the left: negative associations. OTUs on the right: positive associations. The 30 Top OTU markers showing significant association with FRAP measures (with FDR < 0.2) are coloured in green for positively associated and in red for negatively associated markers, respectively. Species-level taxonomic classification of these 30 top markers is shown. **c.** Variation of FRAP measures at W0 and W6 accounting for old and young sub-groups. **d.** Scatter plots showing the subject-specific (W6-W0) changes in glucose levels in Y axis with the corresponding changes in FRAP assay measures, and the mean abundances of FRAP positive and FRAP negative markers in X axis. The Spearman Rhos and the p values for each association are indicated.

Table S 1 P_{adj} values of Wilcoxon Signed Rank tests comparing the abundances of the various taxa in fermenter samples belonging to the various supplementation groups. A: high abundance taxa that are shown in Figure 2; B: low abundant taxa shown in Figure S5.

**(A)**	***Escherichia/Shigella***	***Raoultella***	***Faecalibacterium***	***Bifidobacterium***
ANTH/FLAV	0.4	0.2	0.2	1.0
MIX	0.4	1.0	1.0	0.1
PACs	0.4	0.7	0.1	0.4
S/A	0.7	0.7	1.0	0.7
TPP	0.4	1.0	1.0	0.7
**(B)**	***Phascolarctobacterium***	***Gemmiger***	***Clostridium_XlVb***	***Sutterella***
ANTH/FLAV	0.5	0.75	0.25	0.14
MIX	1	0.25	0.25	0.03
PACs	0.5	0.25	0.25	0.53
S/A	0.5	1	0.5	0.58
TPP	0.5	0.25	1	0.53
	Erysipelotrichaceae	***Parasutterella***	***Flavonifractor***	***Burkholderiales***
ANTH/FLAV	0.5	1	1	0.5
MIX	0.25	0.25	0.75	0.75
PACs	0.25	0.5	0.75	0.75
S/A	0.5	1	1	1
TPP	0.75	0.5	1	0.75
	Oscillibacter			
ANTH/FLAV	1			
MIX	0.25			
PACs	0.25			
S/A	1			
TPP	0.25			

Table S 2 Taxonomic classifications of (A) FRAP Positive and (B) FRAP Negative OTU markers obtained using SPINGO. The confidence score of each affiliation is also indicated.

**(A) FRAP Positive Markers**		
**OTU_ID**	**Species**	**Score**
OTU_100	*Alistipes shahii*	1
OTU_243	*Alkaliphilus crotonatoxidans*	0.03
OTU_458	AMBIGUOUS	0
OTU_146	AMBIGUOUS	0
OTU_523	AMBIGUOUS	0
OTU_826	AMBIGUOUS	0
OTU_575	AMBIGUOUS	0
OTU_68	AMBIGUOUS	0
OTU_124	AMBIGUOUS	0
OTU_317	AMBIGUOUS	0
OTU_354	AMBIGUOUS	0.45
OTU_661	*Anaerostipes hadrus*	1
OTU_50	*Blautia faecis*	1
OTU_220	*Clostridium methylpentosum*	0.22
OTU_134	*Clostridium spiroforme*	1
OTU_344	*Coprococcus catus*	0.77
OTU_557	*Eubacterium hallii*	0.85
OTU_630	*Eubacterium rectale*	0.72
OTU_59	*Eubacterium siraeum*	1
OTU_929	*Faecalibacterium prausnitzii*	0.45
OTU_508	*Faecalibacterium prausnitzii*	0.65
OTU_27	*Gemmiger formicilis*	0.9
OTU_147	*Intestinimonas butyriciproducens*	0.3
OTU_543	*Ruminococcus obeum*	1
OTU_973	*Ruminococcus obeum*	0.42
**(B) FRAP Negative Markers**		

OTU_ID	Species	Score
OTU_15	AMBIGUOUS	0
OTU_226	*Clostridium paraputrificum*	1
OTU_66	*Clostridium ramosum*	1
OTU_22	*Eubacterium cylindroides*	1
OTU_131	*Ruminococcus gnavus*	1
